# Discriminant Analysis Approach in Morphometric Differentiation and Characterization of Serbian Autochthonous Goats

**DOI:** 10.3390/ani13121952

**Published:** 2023-06-10

**Authors:** Nevena Maksimović, Bogdan Cekić, Ivan Ćosić, Dragana Ružić Muslić, Violeta Caro Petrović, Nenad Stojiljković, Nikola Stanišić

**Affiliations:** Institute for Animal Husbandry, Autoput 16, 11080 Belgrade, Serbianikola0135@yahoo.com (N.S.)

**Keywords:** autochthonous goats, morphometrics, discriminant analysis, Serbian White breed

## Abstract

**Simple Summary:**

Goat production is the least popular and most underrated livestock sector in the Republic of Serbia. Back in the 1950′s, goats were anathematized as being the ‘’destroyers’’ of the woods, and in 1954, the infamous Law on the Prohibition of Goat Keeping was implemented on the pretext of preserving the woods. This was unprecedented and never seen anywhere worldwide, but it led to the almost complete devastation of goats as it lasted for decades. Revitalization of the goat sector began decades later through the import of high-yield breeds such as French Alpine and Saanen goats. However, the autochthonous populations of goats that were on the brink of extinction are still to this day considered endangered. There is insufficient scientific data supporting goat production in Serbia, but there’s primarily a lack of relevant research concerning autochthonous breeds. The present study is an attempt to contribute to overall scientific research in the goat sector and particularly to goat genetic resources through their characterization, as recognizing, preserving, and encouraging the breeding of locally specific breeds is important from the standpoint of zootechnical, ethical, cultural, as well as scientific reasons.

**Abstract:**

This study investigated the possibility of using morphometric measurements to differentiate the autochthonous Serbian White goat breed from Saanen and Balkan goats, which were used as sire and dam breeds in its creation. For this purpose, a multivariate discriminant analysis was used. A total of 11 morphometric traits were measured in 98 does of 3 breeds: Saanen (*n* = 28), Balkan (*n* = 28), and Serbian White (*n* = 42), aged 2 to 7 years, in 4 different locations. Univariate analysis of variance revealed a significant difference in body measurements of all three breeds, with Saanen goat being the largest in format and Balkan the smallest. Discriminant analysis extracted six out of eleven tested morphometric traits with the strongest discriminatory power: heart girth, head length, chest depth, head width, pelvic width, and body length. Mahalanobis distances were significant between all three genetic groups. The discriminant function correctly classified 95.24% of the Domestic White goats investigated to their source group. The classification accuracy of the function was cross-validated and indicated an overall success rate of 91.84%. The results of this research showed that there was a clear separation between Serbian White, Saanen, and Balkan goats. The present findings could help a more rapid field assessment of Serbian White goats.

## 1. Introduction

The distinctive sets of genetic diversity amongst animals are represented by the broad span of species and breeds that evolved in varying environments [1]. Therefore, all native animal breeds are an invaluable and irreplaceable source of genetic diversity, as well as a vital cultural and traditional legacy.

Two autochthonous goat breeds are raised in Serbia, the Balkan goat and the Serbian White goat (also known as the Domestic White goat). These breeds are particularly well-suited for breeding in harsh, hilly, remote areas with little vegetation and production systems that almost exclusively rely on the use of natural pastures [2,3] and are mainly raised for combined milk and meat production. The Serbian White goat is a local breed created by crossing autochthonous Balkan and Saanen goat breeds in order to improve milk production in Balkan goats. For this, the Balkan goat was used as a maternal breed, while the Saanen bucks were used as sires. However, the crossing process was nonstrategic, resulting in this new population being unconsolidated (incoherent) in both morphology and productivity [4]. By its phenotype, the Serbian White goat resembles the Saanen breed, but is somewhat smaller, i.e., has a smaller frame size, which is usually due to poor rearing conditions and the absence of proper selection [5]. However, some individuals can be as prominent as pure Saanen goats and can resemble a great deal phenotypically. The coat color of the Serbian White goat is pure white, which is the same as the Saanen goat. The one peculiarity differentiating these breeds in terms of the coat is its length. Serbian White goat has a somewhat longer coat than Saanen and shorter than Balkan. This is usually noticeable along the back line, flanks, and belly. However, this feature is sometimes not obvious, and Serbian White goats can also have short coats like Saanen.

As stated by Lanari et al. [6], the characterization of livestock breeds is the first approach to the sustainable use of animal genetic resources. The characterization of animal genetic resources for food and agriculture is based on three types of information: phenotypic, genetic, and historical [7]. The first step of characterizing autochthonous genetic resources is always based on the knowledge of variation in the morphological traits [8]. Morphometrics refers to the quantitative analysis of form, size, and shape. Morphometric measurements are standard selection tools used routinely in everyday practice, as they are cost-effective and easy to obtain. According to Dossa et al. [9], morphological parameters, including body length, withers height, and heart girth, can be utilized to quickly identify large animals in the field and enable the development of elite flocks. Blackiht and Reyment [10] introduced the idea of “Multivariate Morphometrics,” in which they applied canonical variate analysis, principal component analysis, and related methods, which are commonplace distance measurements between diagnostic features, to standard measurements on organisms such as lengths, heights, and breadths [11]. Many authors have since proposed the use of morphometric traits through means of multifactorial analysis in order to assess phenotypic variation within and between small ruminant populations [1,12,13].

As a representative of the only two autochthonous goat populations in Serbia, the Serbian White goat is very important from the standpoint of genetic resources, and its preservation is of utmost interest. Its status in the DAD-IS FAO database [14] is critically at risk, and its number is very small; therefore, it is essential for every single individual of these animals to be accounted for. Unfortunately, there was no official recognition of the breed in Serbia. The Serbian White goat, or Domestic White goat, as it was initially called, was created through the unplanned crossing of Balkan does with Saanen bucks. This process started before the Second World War in the former county of Yugoslavia, which Serbia was part of, but had to be abandoned when the war started. Years after the war was over, the process of crossing resumed, but then in the mid-1950s, the Law on Prohibition of Goat Breeding was implemented [5]. From that point, all matters concerning goats and goat keeping in the country were performed in secrecy. There was no official monitoring of the crossing process, and by the time of the informal abolition of the law in question, the majority of the work was already complete. At the beginning of the 1990s, another war (the Yugoslav Wars) started in the country, which resulted in the breakdown of Yugoslavia. It took years to revive the livestock sector, as well as goat production, in Serbia after the war. Domestic White goats remained in the Republic of Croatia by the name of Croatian White goat and in the Republic of Serbia under the name of Serbian White goat. It was only recently, i.e., in the past few years, that the work on the conservation of goat genetic resources in Serbia regained some attention and funding. 

Recognizing, preserving, and encouraging the breeding of specific breeds of domestic animals for a specific breeding area and land are measures that must be implemented for zootechnical, ethical, cultural, scientific, and other reasons, and all in order to preserve one’s own identity [15]. Access to a wide variety of animal genetic resources is necessary for the long-term viability of animal production systems and future food security [16]. Animal genetic resources are one of the key components of agricultural biodiversity and thus provide a chance for animal adaptation to changing conditions, especially in light of climate change [17]. Despite its national importance, information on the Serbian goat resources is insufficient, making the characterization of different local genetic types difficult. While genotypic characterization through DNA analysis offers precise identification and affiliation, it is often money-consuming and has practical limitations. Regarding this, the aim of this study was to investigate whether some of the morphometric measurements can be used for quick appropriate field distinction between these breeds, as well as to characterize the Serbian White breed based on morphological variation using multivariate discriminant analyses. The obtained data will help in the proper management, conservation, and genetic improvement of local stock. 

## 2. Materials and Methods

### 2.1. Animals and Location

This study included a total of 98 heads of three goat breeds: Serbian White (42), Saanen (28), and Balkan (28) goats. Serbian White goats were located in central Serbia (villages of Rosica and Trešnja), Saanen goats were located in north-western Serbia (village of Kukujevci), and Balkan goats were found in western Serbia (mountain Rožanj). Each breed originated from a different farm, but within the breed, all animals belonged to one farm, except for the Serbian White goat, where animals originated from two different breeders/farms. All animals included in this study were reproductively mature females, two to seven years old. At the time of this study, no molecular analysis had been carried out on these animals, and the identification was based on phenotypes and the available pedigree information (herd book records). Does of Serbian White and Balkan breed were raised extensively on pastures during the warm period of the year and indoors during cold months, while Saanen does were kept almost exclusively indoors under intensive farm management.

### 2.2. Data Collection

Thirteen morphometric measurements were taken from each animal using a Lydthin stick, tape measure, and Vernier calipers. Measurements were as follows: wither height—measured as the distance from the surface of a platform to the withers; body length—as the distance between the shoulder and pin bone (*tuber ischii*); heart girth—represented the circumference of the chest; chest width—as the width of the rib cage between the fore legs; chest depth—as the vertical distance from sternum to withers; pelvic with—as the distance between the two pelvic bones (*tuber coxae*), across the dorsum; front cannon perimeter—represented the circumference of the left front cannon bone; head length—as the frontal distance from the mouth to poll; head width—measured as maximum distance between zygomatic arches; ear length—distance from the base to the tip of the right ear, along the dorsal surface; and neck girth—as the distance round the mid-region of the neck. All body measurements were estimated by the same two technicians. 

### 2.3. Statistical Analysis

All analyses were performed using the SAS/STAT package ver. 9.4/2013 [18]. Normality of variables was checked with the Shapiro–Wilk test through PROC UNIVARIATE procedure. Least squares means and their standard errors were then calculated for each linear body trait using the PROC GLM procedure in which breed was fitted as a fixed effect. When analysis of variance declared a significant difference, multiple least square means were then compared using Scheffe’s test. Mean, standard deviation, and coefficient of variation were computed for each trait and each breed. Multivariate discriminant analysis was performed to identify the combination of variables that differentiate between the three breeds. Stepwise discriminant analysis was performed using PROC STEPDISC to determine which of the tested measurements had the strongest discriminatory power. Canonical discriminant analysis was performed through the CANDISC procedure to calculate the Mahalanobis distance and canonical coefficient and visually interpret the differences in goats. The ability of canonical functions to assign each animal to its breed was calculated as the percentage of correct assignment to each genetic group (breed) using the DISCRIM procedure. Accuracy of the classification was evaluated using cross-validation. 

## 3. Results

Mean values for eleven morphometric traits and tests of significance among the three breeds of goats are presented in Table 1.

The analysis of variance results obtained by GLM presented in Table 1 showed that breed had a significant effect (*p* < 0.01; *p* < 0.001) for all investigated morphometric parameters except for ear length and front cannon bone perimeter. The coefficients of variation for the different measures showed a generally low variability indicating that the does within each breed were of similar size. Saanen goats had the highest mean values of all measured body traits.

Results of the stepwise discriminant analysis showing Wilks’ lambda values, partial R-squared values, F-values, and probability statistics are presented in Table 2.

As can be seen from the discriminant function analysis summary presented in Table 2, the stepwise discriminant analysis showed that heart girth, head length, chest depth, head width, pelvic width, and body length were the most discriminating variables between Serbian White, Balkan, and Saanen goats. Their partial R2 varied from 0.7289 to 0.0451.

Standardized canonical discriminant function coefficients are presented in Table 3.

The analysis identified two significant canonical axes (*p* < 0.001), CAN1 and CAN2, representing 54.44 and 45.56% of the total variation, respectively. The first discriminant function was weighted most heavily by the head length and chest depth, followed by heart girth. The second function was marked mainly by heart girth and head length, followed by chest depth.

**Figure 1:**
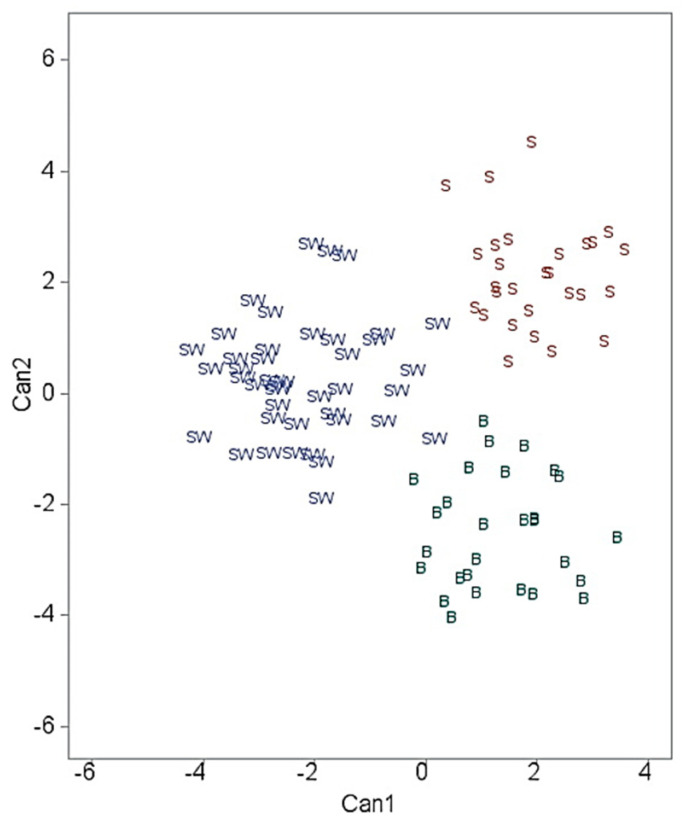
Canonical plotting visually describing goat breed grouping based on linear body traits (symbols: B = Balkan, SW = Serbian White, S = Saanen).

A scatterplot of canonical scores is given in Figure 1.

As shown in Figure 1, all three groups (breeds) were clearly plotted (separated) away from each other. 

The Mahalanobis distance between the three goat breeds is presented in Table 4. All pair-wise distances were highly significant (*p* < 0.001). The distance calculated was somewhat smaller between Serbian White and Balkan goats compared to the distance between Serbian White and Saanen goats. The largest distance was found between Balkan and Saanen goats.

The proportions of correct classification and misclassifications were recorded and are presented in Table 5.

From Table 5, 97.62% of the original observations from the Serbian White goats group were correctly classified, with only one representing the remaining 2.38% being misclassified into the Saanen goat group. Furthermore, 100% of the Saanen and Balkan breeds were correctly classified into their respective group. In total, 98.98% correct classification was achieved through discriminant function with six traits. The table also summarises the results of cross-validated analysis. In all, approximately 91.84% correct classification rate was achieved under the cross-validated results, with 95.24% correct classification for the Serbian White goat, as well as 89.29% for both Saanen and Balkan goats. 

## 4. Discussion

In livestock breeding, the most common way to describe the breed is the description of its external markings, measurement of body characteristics, and production traits [19]. Morphometric measurements are routinely used in everyday practice to provide information for selection purposes. Different studies reported a strong correlation between some morphometric measurements and production traits [20,21,22,23]. Moreover, linear body measurements are important data sources for portraying breed standards. Phenotypic characterization can often be challenging due to the existence of many populations that are not assigned to any recognized breed or are very similar to each other, being the result of multiple crossings of recognized breeds [7]. The Serbian White goat is one of those populations, resulting from crossing Balkan goats, which is a broad term used for the goats found around the Balkan Peninsula and Saanen bucks. Based on most of the measured traits in the present study, the Serbian White goat is smaller than the Saanen goat in body format and somewhat larger than Balkan. This is expected because the Serbian White goat represents the cross between the other two breeds. However, its body format is somewhat closer to the Balkan goat, which could mean that the Serbian White goat is either genetically closer to the Balkan goat or that the low level of selection and poor rearing conditions did not allow for a full expression of body format development. The coefficients of variation for the different measures showed a generally low variability indicating that the does from within each breed had similar sizes. The least-squares variance analysis results of the present study indicated that breed had a significant effect (*p* < 0.01; *p* < 0.001) for all investigated parameters except for ear length and front cannon bone perimeter. Morphometric body measurements of the Serbian White goat in the present study varied from the data presented by Antunović et al. [24] for the Croatian White goat, which is the same breed raised in Croatia under a different name. The authors reported lower measurements of wither height 64.29 cm, body length 65.24 cm, and chest depth 27.63 cm and higher measurements for chest width 16.24 cm, heart girth 83.34 cm, and cannon bone perimeter 8.66 cm. Measured body traits of Balkan goats for whiter height, body length, heart girth, chest with, chest depth, and cannon bone parameters were lower than data previously published by Mioč et al. [25] for Balkan goats raised in Croatia (called Croatian colored goat). Memiši et al. [26] also stated higher values for specific body measurements of Balkan goats: wither height 66 cm, body length 70 cm, chest depth 30 cm, and chest width and hip width 17 cm each, on average. However, these studies do not state how exactly the measurements were taken, and different measuring points can have a profound impact on obtained values, especially for measurements of pelvic/hip width and chest width as well. 

Discriminant analysis of morphometric traits is broadly used in determining the relationships among different breeds of livestock [27,28,29]. Sanni et al. [30] proposed that more than three phenotypic variables should be included in order to minimize ambiguity in classification when assessing morphological diversity. In the present study, eleven morphometric variables were stepwise introduced as predictor variables into the discriminant analysis. The stepwise discriminant analysis showed that six traits, including heart girth, head length, chest depth, head width, pelvic width, and body length, were the most discriminating variables between Serbian White, Balkan, and Saanen goats. All other variables have been removed from the final model. Similarly, Hilal et al. [31] found head length, head width, pelvic width, and chest width to be the most discriminant variables between the two populations of Moroccan Hamra goats raised in two different regions. Due to the joint consideration of all measured morphological variables, multifactorial analyses of morphological traits are more appropriate for assessing phenotypic variation within and between populations as well as appropriately discriminating between different goat breeds [9,32]. 

Wilks’ lambda is an inverse measure of the prominence of the discriminant functions as it represents the ratio of the within-group variability to the total variability of discriminant variables [33]. The value of the Wilks’ lambda in the present study was 0.0529, i.e., 5.29%, suggesting that almost all the variability (the rest 94.71%) comes from the differences between investigated populations (breeds) instead of within the population. The diversity between breeds is a notable criterion to look for in the conservation of animal genetic resources in any genetic development program [34]. 

Canonical analysis was used to obtain the function of all body measurements necessary for the separation of the three breeds. The standardized canonical coefficients indicate each variable’s partial contribution to the discriminant function [29]. The first discriminant function was weighted most heavily by the head length and chest depth, followed by heart girth. The second function was marked mainly by variables heart girth and head length, followed by chest depth. Thus, measurements of the head and chest had an important contribution to both functions. Both functions were found statistically significant, although the first function had a slightly higher proportion of explained variance, with 54.44% of all discriminatory power explained by this function vs. 45.56 for the second function. On the contrary, Selolo et al. [35] reported a higher value for the first canonical variable that accounted for almost all the variation (91.9%) for South African indigenous goats. Similar to the present study, in the studies of Ogah [36], the distribution of discriminatory power among the two functions was more balanced, as CAN1 and CAN2 accounted for 59.7 and 40.3 % of the total variation in chickens.

The Mahalanobis distance was estimated from the mean values of six body measurements selected by stepwise discriminant analysis. The significant pairwise distances showed the existence of measurable group differences between the investigated goat breeds, suggesting that the breeds were morphologically different. The distance calculated was somewhat smaller between Serbian White and Balkan goats compared to the distance between Serbian White and Saanen goat breeds. The largest distance was found between Balkan and Saanen goats, which is expected given the fact that the two are entirely genetically and morphologically different breeds. Yakubu et al. [27] also reported significant Mahalanobis distance between West African Dwarf and Red Sokoto goat breeds, thus indicating considerable genetic variation between these breeds. Unlike this study, Hilal et al. [31] found the Mahalanobis distance between the two populations of Hamra goats of Beni Arouss and Rommani regions to be very low, indicating a morphological similarity between the two populations. They argue that this similarity may be due to two reasons: the genetic exchange that might have taken place between the two populations in the past or because of a common or close origin. Similarly, comparing three Saudi goat types, Ardi (which is considered a definite breed) and Lines 1 and 2 goats (which have been developed from unknown origins), Aziz and Al-Hur [37] found that based on the Mahalanobis distance between Lines 1 and 2, both lines were more closely related to each other than to Ardi breed. These suggest that Mhalanobis distance can be considered a reliable distance measure for quantitative traits of livestock breeds.

The ability of canonical functions to assign each individual animal to its breed was calculated as the percentage of correct assignment to each genetic group (breed). The discriminant function was able to correctly classify 95.24% of the Serbian White goats investigated using cross-validation. Only two out of forty-two Serbian White does were misclassified into the Saanen and Balkan groups (one in each group). All the individual Saanen and Balkan goats were originally correctly allocated to their genetic groups (100%), although cross-validation showed a less accurate classification of 89.29% each. In total, 98.98% correct classification was achieved from the original count, as well as 91.84% under the cross-validated results. Similarly, 98% overall correct classification was achieved by Melesse et al. [34] in the study of morphological characterization of the indigenous goat population in Ethiopia. Yakubu et al. [27] correctly allocated 99.4% and 100% of individual goats into their source genetic groups and achieved a 99.7% success rate under the cross-validated results. Asamoah-Boaheng and Sam [28] achieved approximately 82.0% correct classification of sheep breeds with quadratic discriminant function as well as 86.9% correct classification rate under the cross-validated results.

For the practical field assessment and in cases when a molecular analysis is not available for the classification of Serbian White goats, morphometric measurements of heart girth, head length, chest depth, head width, pelvic width, and body length can be used alongside phenotypic (visual) characteristics. As the discriminant analysis showed measurements of the head and chest to have an important contribution to both canonical functions, these traits should be considered as the most explanatory.

## 5. Conclusions

This study showed that head length, chest depth, head width, heart girth, pelvic width and body length were the most discriminating variables to separate Domestic white from Saanen and Balkan goats. The present findings could help with a more rapid field assessment of Serbian White goats and could also be valuable for reinforcement of the traditional in situ conservation programs of autochthonous goat genetic resources for their sustainable implementation. The classification accuracy obtained was very high, even considering the fact that these are all post hoc classifications. However, if correct predictive classification is the goal of the research, then at least two studies must be conducted: one in order to build the classification functions and another to validate them. The current study demonstrated that multivariate analysis could be used to characterize native goat breeds based on morphological traits in cases where molecular tools are unavailable. Moreover, additional molecular analyses that would complement the phenotypic differences discovered in this work could help preserve and advance autochthonous breeds through suitable selection and breeding programs.

## Figures and Tables

**Table 1:** Least square means and descriptive statistics of the morphometric traits of Serbian White, Saanen, and Balkan goats.

Traits/Breed	Serbian White			Saanen			Balkan		
	Mean ± SE	SD	CV	Mean ± SE	SD	CV	Mean ± SE	SD	CV
Wither height ***	66.95 ± 0.59 b	4.1	6.12	74.83 ± 0.71 a	3.0	4.0	64.25 ± 0.72 b	4.06	6.32
Body length ***	71.45 ± 0.66 b	4.88	6.83	80.79 ± 0.8 a	3.61	4.46	65.25 ± 0.81 c	4.06	6.22
Heart girth ***	78.86 ± 0.69 b	4.81	6.73	90.84 ± 0.85 a	4.21	4.63	72 ± 0.85 c	4.21	5.85
Chest width ***	13.82 ± 0.26 b	1.83	13.24	17.14 ± 0.31 a	1.88	10.97	13.18 ± 0.32 b	1.25	9.48
Chest depth ***	29.31 ± 0.28 b	1.84	6.28	32.03 ± 0.34 a	1.57	4.9	25.32 ± 0.35 c	2.11	8.33
Pelvic with ***	10.39 ± 0.16 b	1.06	10.2	12.93 ± 0.19 a	0.88	6.8	10.5 ± 0.19 b	1.07	10.2
Front cannon perimeter	7.53 ± 0.9	0.54	7.17	8.52 ± 1.08	0.53	6.22	7.82 ± 1.1	0.58	7.41
Head length ***	22.27 ± 0.34 b	1.92	8.62	29.40 ± 0.41 a	2.28	7.75	28.35 ± 0.41 a	2.48	8.74
Head width **	12.62 ± 0.19 b	1.59	12.59	15.52 ± 0.23 a	0.99	6.39	13.55 ± 0.23 c	0.64	4.72
Ear length	13.77 ± 0.32	2.13	15.46	14.83 ± 0.38	1.04	7.01	14.30 ± 0.39	2.62	19.69
Neck girth ***	28.46 ± 0.39 b	2.9	10.19	33.6 ± 0.46 a	1.66	4.94	28.8 ± 0.47 b	2.51	8.71

SE—standard error; SD—standard deviation; CV—coefficient of variation; ^a,b,c^—row means with different letters differ significantly; *** *p* < 0.001, ** *p* < 0.01.

**Table 2:** Morphometric characters selected by stepwise discriminant analysis to separate three analyzed sheep breeds—discriminant function analysis summary.

Variables	Partial R^2^	F-Value	Pr > F	Wilks’ Lambda	Pr < Lambda	Average Squared Canonical Correlation	Pr > ASCC
Heart girth	0.7289	127.71	<0.0001	0.27109850	<0.0001	0.36445075	<0.0001
Head length	0.6961	107.64	<0.0001	0.08239721	<0.0001	0.71245785	<0.0001
Chest depth	0.1786	10.11	0.0001	0.06768158	<0.0001	0.73953891	<0.0001
Head width	0.1297	6.86	0.0017	0.05890111	<0.0001	0.75646622	<0.0001
Pelvic width	0.0588	2.84	0.0636	0.05543998	<0.0001	0.76346342	<0.0001
Body length	0.0451	2.13	0.1253	0.05293954	<0.0001	0.76917766	<0.0001

**Table 3:** Standardized canonical discriminant function coefficients.

Variables	F1 (CAN1)	F2 (CAN2)
Heart girth	0.739427796	0.924752407
Head length	1.546429812	−0.767958964
Chest depth	−1.201890200	0.438036686
Head width	0.550726573	0.193068906
Pelvic width	0.300067735	0.300884115
Body length	0.150132568	0.347013475
Eigenvalue	3.8366	3.2108
Cumulative	0.5444	1.0000
% of variance	54.44	45.56
Canonical Correlation	0.890641	0.873222

**Table 4:** Mahalanobis distance * value and probability of significance between Serbian White, Saanen, and Balkan goat breeds.

Breed	Serbian White	Saanen	Balkan
Serbian White	0.0000	21.01101	20.21202
Saanen	<0.001	0.00000	21.85317
Balkan	<0.001	<0.001	0.0000

* Values above the diagonal show the value of Mahalanobis distance; values below the diagonal show the probability of significance of Mahalanobis distance.

**Table 5:** Classification matrix (percent (%) of individual goats classified into breed).

Breed	Percent %	Serbian White	Saanen	Balkan
Original				
SW	97.62	41	1	0
S	100	0	28	0
B	100	0	0	28
Total	98.98	41	29	28
Priors		0.3333	0.3333	0.3333
Cross-validated				
SW	95.24	40	1	1
S	89.29	3	25	0
B	89.29	2	1	25
Total	91.84	45	27	26

## Data Availability

Not applicable.
